# Why should we apply ABM for decision analysis for infectious diseases?—An example for dengue interventions

**DOI:** 10.1371/journal.pone.0221564

**Published:** 2019-08-27

**Authors:** Florian Miksch, Beate Jahn, Kurt Junshean Espinosa, Jagpreet Chhatwal, Uwe Siebert, Nikolas Popper

**Affiliations:** 1 dwh Gmbh, Vienna, Austria; 2 Department of Computer Science, University of the Philippines Cebu, Cebu City, Philippines; 3 Institute of Public Health, Medical Decision Making and Health Technology Assessment, Department of Public Health, Health Services Research and Health Technology Assessment, UMIT-University for Health Sciences, Medical Informatics and Technology, Hall in Tirol, Austria; 4 Institute for Technology Assessment and Department of Radiology, Massachusetts General Hospital, Harvard Medical School, Boston, Massachusetts, United States of America; 5 Division of Health Technology Assessment and Bioinformatics, ONCOTYROL - Center for Personalized Cancer Medicine, Innsbruck, Austria; 6 Center for Health Decision Science, Department of Health Policy and Management, Harvard T.H. Chan School of Public Health, Boston, Massachusetts, United States of America; Fundacao Oswaldo Cruz, BRAZIL

## Abstract

For the evaluation of infectious-diseases interventions, the transmissible nature of such diseases plays a central role. Agent-based models (ABM) allow for dynamic transmission modeling but publications are limited. We aim to provide an overview of important characteristics of ABM for decision-analytic modeling of infectious diseases. A case study of dengue epidemics illustrates model characteristics, conceptualization, calibration and model analysis. First, major characteristics of ABM are outlined and discussed based on ISPOR and ISPOR-SMDM Good Practice guidelines. Second, in our case study, we modeled a dengue outbreak in Cebu City (Philippines) to assess the impact interventions to control the relative growth of the mosquito population. Model outcomes include prevalence and incidence of infected persons. The modular ABM simulates persons and mosquitoes over an annual time horizon considering daily time steps. The model was calibrated and validated. ABM is a dynamic, individual-level modeling approach that is capable to reproduce direct and indirect effects of interventions for infectious diseases. The ability to replicate emerging behavior and to include human behavior or the behavior of other agents is a distinguishing modeling characteristic (e.g., compared to Markov models). Modeling behavior may, however, require extensive calibration and validation. The analyzed hypothetical effectiveness of dengue interventions showed that a reduced human-mosquito ratio of 1:2.5 during rainy seasons leads already to a substantial decrease of infected persons. ABM can support decision-analyses for infectious diseases including disease dynamics, emerging behavior, and providing a high level of reusability due to modularity.

## Introduction

Decision analysis is a systematic approach to decision making under uncertainty [1]. The application of decision-analytic models (i.e. simulation models) allows the evaluation of new technologies (e.g., treatments, vaccinations, interventions against the spread of the disease) with respect to benefits, risks, harms and costs. Such computer simulation models are necessary where randomized controlled clinical trials or observational studies are missing, or not feasible [2]. Decision-analytic models (DAM) synthesize available information from various sources (e.g., epidemiological studies on the natural history of the disease or the spread of the disease, short-term clinical studies on treatment effects, quality of life studies, expert opinions) for their short- and long-term evaluations [3].

For the evaluation of interventions in the area of infectious diseases, the transmissible nature of infectious diseases plays a central role when developing a model. The reduction of the prevalence of infections in the target population often leads to a decreased risk for other individuals in the community. As a best case, the infectious disease may even be eliminated if the number of infected individuals decreases below a critical number. It is therefore paramount for decision-analytic models to capture not only the direct effect of interventions on the individuals under intervention but also indirect effects on the population level including herd immunity [4–7]. Herd immunity refers to the effect that a partly vaccinated population can prevent the spread of an infectious disease, and thereby protect unvaccinated persons. In addition, the possibility of acquiring natural immunity following recovery from infection should be considered.

The recent guidelines of the ISPOR-SMDM Modeling Good Research Practices Task Force [4, 8, 9] note that some modeling methods for decision-analytic models in communicable diseases, such as Markov state-transition cohort models, ignore the indirect effects that arise from averted incident infections, and therefore benefits and cost-savings from the intervention may be underestimated [2, 7]. In the review of Kim et al. [10], the authors summarize that model choice for cost-effectiveness analyses for vaccination programs need to be improved, for instance, to capture the effect of herd immunity. Jit and Brisson [5] state that many published decision-analytic modeling studies in infectious diseases do not take into account the specific features of infectious diseases such as the transmissibility from infected to susceptible individuals and the uncertainties arising from complex natural history and epidemiology.

In our article, we will illustrate methodological issues in disease transmission modeling using dengue fever as an exemplary infectious disease. Dengue is a mosquito-borne tropical infectious disease caused by the dengue virus. The dengue virus is transmitted by several species of mosquitoes thriving in tropical countries, principally by *Aedes aegypti* [11, 12]. Local and regional dengue outbreaks are observed in many tropical countries, and increased number of cases is observed during rainy season [13]. Dengue resurged in the 20th century and received public awareness in the past few decades when epidemics became stronger and more severe [14]. With now half of the world’s population being at risk, dengue epidemics are a severe public health problem in tropical countries, causing a large and continuing morbidity and mortality burden. The Global Burden of Disease Study 2013 estimated a total of 576,900 (330,000–701,200) years of life lost to premature mortality attributable to dengue in 2013 and dengue was responsible for 1.14 million disability-adjusted life-years in 2013 [15, 16].

To model dengue epidemics, several modeling approaches have been applied. For example, differential equations models are used to predict the outcome of vaccinations [17] or to analyze epidemiologic aspects [18, 19]. However, differential equations models are inflexible because incorporation of details would require a high (and potentially unmanageable) number of disease compartments. Sitepu et al [20] predicted dengue epidemics using a statistical autoregressive model. However, such statistical methods are limited to specific scenarios and do not incorporate dynamic effects. Several compartment and stochastic simulation models have been applied to predict the impact and cost-effectiveness of Dengvaxia, the first available dengue vaccine [21]. Hladish et al presents two studies of agent-based models of humans and mosquitoes for dengue transmission [22, 23]. A recent review of health economic evaluation studies of dengue vaccines found that out of 13 studies, 8 studies used dynamic mathematical models of dengue transmission, and 2 studies used a Markov model [24].

For evaluations of the effect of interventions on disease spread, the international guidelines developed by the ISPOR-SMDM Modeling Good Research Practices Task Force recommend modeling methods that capture interaction, such as dynamic transmission models [25]. Dynamic transmission modeling is often performed using system dynamics. System dynamics models are deterministic and based on compartments. However, these deterministic compartment models cannot capture stochastic effects such as the extinction of the disease in small populations and complex interactions and behavior. Agent-based models (ABM) are a more flexible alternative approach for dynamic transmission modeling because AMBs are modeling individuals rather than compartments [4].

The aim of our study is, therefore, to discuss the concepts and important characteristics of ABM for decision-analytic modeling of infectious diseases. In a case study of dengue epidemics, we apply an ABM model to analyze the impact of potential efficacy of mosquito control interventions on the number of new dengue infections in humans in the Philippines. The case study serves to illustrate model characteristics, conceptualizing the model, experiences with model calibration and model analysis.

## Case example dengue

Dengue is a mosquito-borne tropical disease that can result in a fever with a mild to life-threatening progression [26–28]. There are five serotypes of dengue virus [29]. After an infection, a person is immune against the specific serotype. However, studies show that further infections with other serotypes often result in more severe disease progressions [12, 30].

In the Philippines, dengue is prevalent for the entire year with a small number of infections during dry season and significantly higher infection numbers during the rainy season [13]. The prevalence of dengue infections in the Philippines also differs among regions. However, the underlying mechanisms of dengue epidemics are not yet completely understood. Why an outbreak happened at a certain place and time is hard to explain.

Measures against the spread of dengue can be categorized into three types of interventions: (1) prevention from being bitten by mosquitoes (e.g., by repellents), (2) vector control, such as releasing Wolbachia infected or genetically modified mosquitoes, sterilizing techniques, and mass-trapping leading to a reduced number of mosquitoes [31], and (3) vaccinations. The first two measures have been the default interventions for many years. To date, vaccines have been developed and tested in long clinical trials and are now publicly available in selected countries since early 2016 [13, 32, 33].

Within our project, we aimed building an agent-based model which is suitable to simulate a dengue outbreak in a Philippine city or region. The model allows analyzing the mechanisms of dengue epidemics and evaluating the impact of mosquito (vector) control interventions on disease burden. In this paper, we evaluated the impact of human mosquito ratios on disease incidence to show the effectiveness that a future mosquito control should reach to stop the epidemics. Before we describe the model in detail, we summarize and discuss characteristics of ABM.

## Characteristics of ABM and reasons for model selection

Agent-based modeling focuses on complex systems composed of ‘agents’ that are uniquely identifiable, autonomously acting, and capable of interactions [34–36]. Agents are often human individuals but can also be animals, or objects such as vehicles, buildings etc.. These agents can capture population heterogeneity. The agents’ behaviors are often described by simple rules, for example, contacts within social networks or contacts with mosquitos. Agents’ behaviors in combination with transmission pattern and disease progression result in emerging population-wide dynamics such as the outbreak of a disease [37]. In the following, the main characteristics of ABM are discussed with the focus on infectious diseases and the case example of a dengue fever epidemic.

### Dynamic (compared to static)

In transmission models, the incidence of infection is determined by the number of susceptibles and the rate at which susceptible become infected (force of infection). In a static model, the force of infection is constant over time or it can depend on age. In a dynamic model, the force of infection is not a fixed parameter. ABM is a dynamic modeling approach since the probability of an individual acquiring an infection can be implemented depending on contact patterns of the agents (i.e., direct interactions), transmissibility of the infection and the distribution of the infection among the agent population. As a consequence of these factors, the force of infection is usually not constant over time. For example, dengue epidemics mostly underlie seasonal patterns with outbreaks during rainy season. In the dengue model, the mosquito population grows rapidly in a rainy season and more mosquitoes transmit the disease (transmissibility increases), which causes an outbreak. An important advantage, dynamic transmission models are capable of reproducing the direct and indirect effects of interventions for communicable diseases, including herd immunity [4, 10, 34]. Often, dynamic transmission models are used to assess open target populations. However, modeling studies could also assess epidemics in closed populations. Therefore, the terms “dynamic”and “open” are discussed separately.

### Stochastic (compared to deterministic)

In a deterministic model (e.g., system dynamics models), a given initial situation (or set of parameter values) always leads to the same results [38]. In a stochastic model, multiple runs with different underlying random numbers provide a different result due to randomness in model parameters. That is, some model parameters are specified by underlying parameter distributions, and in each run new parameter values are drawn from the distributions. Agent-based modeling is a stochastic and individual-level simulation technique, and therefore, is able to integrate first-order uncertainty. First order uncertainty means that, for example, individuals with the same characteristics (and therefore facing the same probabilities and outcomes) can just by chance experience different effects of a disease [39]. Stochasticity is included through pseudo-random numbers. Running stochastic models and applying different random numbers leads to different model results. In communicable diseases, stochastic effects are particularly relevant, as they can help to model the extinction of a specific disease in small populations, where extinction also depends on chance [4, 5]. Deterministic dynamic transmission modeling methods are not able to capture such small population effects.

### Individual based (compared to aggregated cohorts)

ABM are individual-based models that allow researchers to model explicitly the agents’ heterogeneity regarding specific characteristics including risk factors or behavioral attributes. Risks depending on past events can be incorporated using individual characteristics of agents instead of modeling many compartments as in dynamic transmission cohort models. Modeling individual attributes that affect their behavior explicitly has a great advantage: model adaptation becomes easier. Altering or adding attributes generally does not affect the model structure, as it would be the case if information aboutattributes were captured in compartments in system dynamics models.

In ABM, agents can act independently in their environment, interact with each other, influence each other and learn from their experiences and adapt [4, 37]. ABM can capture non-linear behavior and interactions (i.e., change in outcome that is not proportional to a change in input). The evaluated individual-level (health) outcomes are finally aggregated for health-care decision making.

In our individual-level ABM, dengue epidemics are rapidly evolving and the mosquito population is changing fast due to a short lifespan, requiring detailed states of humans and mosquitoes for age, infection, incubation, viraemia, and recovery. Implementing multiple characteristics and disease history can easily be achieved for simulated individuals through combination of attributes. Such a complexity would require hundreds of compartments (i.e., for all combinations of attributes) and thousands of equations when using different aggregated compartments.

### Open (compared to closed)

ABM makes it possible to model open cohorts, that is individuals can enter into and exit from the model population. Therefore, demographic processes such as birth, death, emigration and immigration can be incorporated. This characteristic is important, for example, to capture seasonal effects of the mosquito population or vertical transmissions of diseases [38].

Mosquitoes have a short lifespan of several days. Old mosquitoes are often infected while newborn mosquitoes are susceptible. This dynamically changing fractions of susceptible and infected mosquitoes cannot be achieved with a closed cohort of mosquitoes.

### Emerging behavior

Emergent behavior “also known as emergence, refers to the novel and coherent structures, patterns, and properties that arise from the interaction of the parts of a complex system and take place at the system scale rather than at the component’s” [40, 41]. ABM is most suited to problems focused on how individual interactions (e.g., contact pattern) generate emergent system behaviors and structures (e.g., potential intended and unintended consequences of an epidemic such as herd immunity, serotype shift, and extinction). In the model, the dengue epidemic is not modeled directly, but is a result of mosquitoes biting humans and the ability to transmit the virus in both directions.

### Network structure, regionality (locality)

An agent’s neighborhood is a general concept. The concept is applicable to various agent spaces such as geographical space or social space specified by the agent’s social network. Agents interact with their environment including other agents [37]. How detailed the environment is modeled (e.g., spatial location of an agent relative to other agents or using rich set of geographic information) depends on the research question.

## Applied ABM for dengue

In this section, we describe the framework, structure, parameters, calibration, validation of the ABM we developed for the evaluation of dengue interventions to control dengue, and the analytic techniques of the evaluation. A detailed model description is provided in the supplementary material S5 File. The model has been implemented in Java SE 8.The program code will be provided upon request.

### Model framework

In this case study, we aimed to examine the 2010 dengue outbreak in Cebu City in the Philippines. We assessed the impact of potential effectiveness of interventions to control the relative growth of the mosquito population on the number of new dengue infections in humans over time. We simulated dengue for a one-year time horizon throughout the whole year 2010 in Cebu City with a population of 860,942 people. The model is based on a recently published theoretical framework using a modular structure [42]. Our ABM consists of two types of agents: persons and mosquitoes that are simulated over an annual time horizon considering daily time steps. Hypothetical efficacy of interventions was modeled in scenarios that reduce the human mosquito ratio to 1:3.0, 1:2.5, 1:2, 1:1.5 and 1:1 during rainy season. Reported model outcomes are prevalence and incidence of infected humans over time. All results for humans can be further stratified by age, gender, and status of the person (susceptible, infectious, and resistant).

### Modular model structure

Our dengue model consist of three modules: (1) population module, (2) contact module, (3) disease modules [42]. This modular structure has been developed to build a complex agent-base model that provides the flexibility of model adaptions, validation and transparency, meaning easier to review. The modules are loosely coupled; that is, modules are designed in the same way, so that strong dependencies are within one module. This reduces dependencies between modules, so that modules can be extended independently and can even be reused in other models. Modules also fully cover a specific area. This strategy allows for developing, implementing, testing and validating the modules separately. This strategy also supports model extensions and adaptations as they usually address a single module rather the entire model. Validated modules can be reused in several projects to increase credibility and decrease development time. This modular structure consists of a population module, a contact module and a disease module.

The population module of the model initializes a population of persons with age and gender representing the demographics of the simulated region or city. In our model, the size of the human population does not change within the simulated one-year time horizon. The population module also initializes mosquitoes including age and gender attributes. The number of mosquitoes is given as a multiplier of the human population. This multiplier can change at specific points in time and is constant otherwise. A mosquito dies every day with a constant probability, or at the maximum age of 33 days, which results in an exponentially distributed age distribution. The number of mosquitoes born each day depends on the number of mosquitoes that died and the defined multiplier. When the multiplier is changed, a minimum or maximum number of newborn mosquitoes can be applied to prevent an abrupt decline or increase of the mosquito population.

The contact module handles the spread of dengue virus due to mosquito bites. Other contacts, for example, person-to-person interactions are not relevant for dengue. Only female mosquitoes bite, following a sophisticated process based on their gonotrophic cycle and the need to suck a certain amount of blood. In short, mosquitoes need to bite several persons each day to get a predefined amount of blood for a certain amount of time defined as biting time. When they reach a certain amount of blood, they can lay eggs again. The time between laying eggs, comprising the time of biting and collecting blood, is called gonotrophic cycle. The mosquitos choose their victims randomly among all persons.

The disease module describes the relevant health states and the disease progressions. It has been observed that a dengue outbreak is usually caused by a single serotype [43]. There is no information about serotype-specific prior infections in the Philippines [11]. Therefore, we model only a single serotype and neglect prior infections. A transmission is possible in both directions with a given probability whenever a mosquito bites a human. However, the host must be in a viraemic state that allows spreading of the virus, and the recipient must be susceptible to the virus. The infection process in mosquitoes is well documented: mosquitoes are usually born healthy and susceptible, although transmissions from infected mothers to the offspring, so-called vertical transmissions, could be observed in rare cases [44, 45]. Upon transmission, a mosquito becomes infectious after an incubation period [30]. Dengue is not harmful to mosquitoes; they simply remain infectious until they die. Humans are also susceptible at simulation start. Upon a transmission, two independent processes start. The first process makes them infectious after an incubation period, until they recover and become resistant to the virus [12, 30]. The second process models the type and length of symptoms after another incubation period. The symptoms range from an asymptomatic disease to dengue fever (DF), severe hemorrhagic fever (DHF), and to life-threatening septic shock (DSS) [26–28].

Once implemented, the model can be simulated; humans and mosquitoes behave as defined, resulting in an epidemic behavior of transmissions, infections and recoveries. Herd immunity is automatically considered as a result of agent’s interactions and transmissions.

### Model parameter and data

To simulate the dengue epidemic in Cebu City, hospital data containing all hospitalized dengue cases in 2010 were applied. The datasets were provided by the City Health office in Cebu City for use in this project (S1 File). The human population was defined according to the Cebu City population of 2010, based on data of the Philippine Census [46]. All model parameters are listed in Table 1, the computations are presented in S2 File. Parameters derived by calibration are marked accordingly. The calibration process is described thereafter.

**Table 1:** Model parameters of the dengue model and sources.

Parameter	Value	Source
Simulation time	357 days	2010 has 51 full calendar weeks (= 357 days)
Rainy season	Day 131 –day 220	Found through calibration
Population and age distribution (humans)	According to demography in Cebu City 2010 with a population of 860,942	Philippine Census of 2010 [46]
Maximum age (humans)	99 years	Requirement due do missing data above age 99
Mosquitoes per human	Dry season: 1.0, rainy season: 3.5	Found through calibration
Maximum age (mosquitoes)	33 days	Based on Southwood et al. [49] (a maximum age of 33 allows a mosquito to live for 34 full days, this is the mean of 30–38 days)
Gonotrophic cycle length (mosquitoes)	4 days	Wong et al. [47]
Mosquito death probability per day (mosquitoes)	0.2	Calibration
Biting time per gonotrophic cycle (mosquitoes)	200–1400 seconds (uniformly distributed)	Platt et. al. [48]
Time per bite (mosquitoes)	5–90 seconds (uniformly distributed)	Expert opinion
Initially viraemic (humans)	0.000533	Based on dengue case data in the first week of 2010 for all patients that are residents of Cebu City
Initially resistant (humans)	0	Assumption
Initially infected (mosquitoes)	0.001	Calibration
Probability of transmission (from mosquitoes to humans)	0.14	Calibration
Probability of reported transmissions (humans)	0.0976	Based on the assumption of 80% asymptomatic cases (arbitrarily chosen from diverging data in Chastel [27]) and that 25% of DF cases and all DHF and DSS cases are reported in the hospital data
Length of incubation period (humans)	5–7 days (random)	McBride et al. [30]
Length of viraemic phase (humans)	4–5 days (random)	Gubler [50]
Length of intrinsic incubation period (humans)	3–10 days (random)	Chan and Johansson [26]
Length of a fever (humans)	2–7 days (random)	Gubler [50]
Probability for type of fever (humans)	DF: 0.3564 DHF: 0.6335 DSS: 0.0102	Based on dengue case data of 2010 for all patients that are residents of Cebu City
Probability of transmission (from humans to mosquitoes)	0.3	Calibration
Length of incubation period (mosquitoes)	8–12 days (random)	McBride et al. [30]

DF: dengue fever, DHF: hemorrhagic fever, DSS: septic shock

The provided datasets about dengue cases were anonymized and did not include any identifiable information. The population data from the Philippine Census is aggregated and does not allow to identify persons. The agents in the model are objects representing aggregated data and do not relate to existing persons.

### Calibration

The human population and the reported dengue cases are well known. However, in addition to the reported cases, there are mild unreported cases and asymptomatic cases. These persons are unknown but they are infectious and can transmit the disease. The number of asymptomatic and unreported dengue cases were estimated based on Chastel et.al. [27].

There is no reliable information about the mosquito population. This includes the population size, lifespan, reproduction numbers, and biting habits. There is some information about incubation period of mosquitoes and that they remain infectious for the rest of their lives. The infection probabilities for humans and mosquitoes are also unknown and are subject for calibration.

The model has been calibrated to the 2010 dengue epidemic in Cebu City for eight unknown model parameters (probability of transmission for persons and mosquitoes, mosquitoes per person during rainy and dry season, mosquito death probability per day, initially infected mosquitoes, and start and end of the rainy season). The epidemic is defined as newly infected persons per week over time based on the hospital case data and assumptions about asymptomatic and unreported cases (Table 1). The calibration goal is to reproduce the incidence numbers.

A manual calibration has been performed because an automated calibration was not possible due to the large number of parameter combinations and time intervals and relatively long simulation times. Calibration results were plotted in order to identify the parameter set that provides the best fit.

The calibration has been performed considering three phases: (1) the situation before the outbreak, (2) the start of the rainy season, and (3) the end of the epidemic. The first calibration phase showed that there is a strong relation between mosquito numbers, biting rates and transmission probabilities. For example, more mosquitoes with less biting rates result in the same human infections. We found some information about biting times of female mosquitoes [47, 48], assumed a uniformly distributed biting time of 5 to 90 seconds and a mosquito population as large as the human population (referred to as “mosquitoes per human” and as a ratio “humans:mosquitoes”), and we assumed that all mosquitoes die at the age of 33 days. The calibration of the first 25 weeks of the year is straight-forward and leads to transmission probabilities for humans and mosquitoes.

In the second calibration phase, first only the number of mosquitoes at week 25 was increased to create an outbreak. Consequently, the epidemic starts as required. The earlier assumed predefined fixed life span of mosquitos of 33 days, however, leads after 33 days to a large number of infectious mosquitoes dying, which are replaced by susceptible newborn mosquitoes. This causes a significant decline in the human epidemic until a significant portion of newborn mosquitoes get infected and pass their incubation period. Therefore, daily dying probabilities and population growth rates were introduced to gain better mosquito demographics. Parameters were adapted until the epidemics between week 25 and 37 were satisfying.

**Fig 1:**
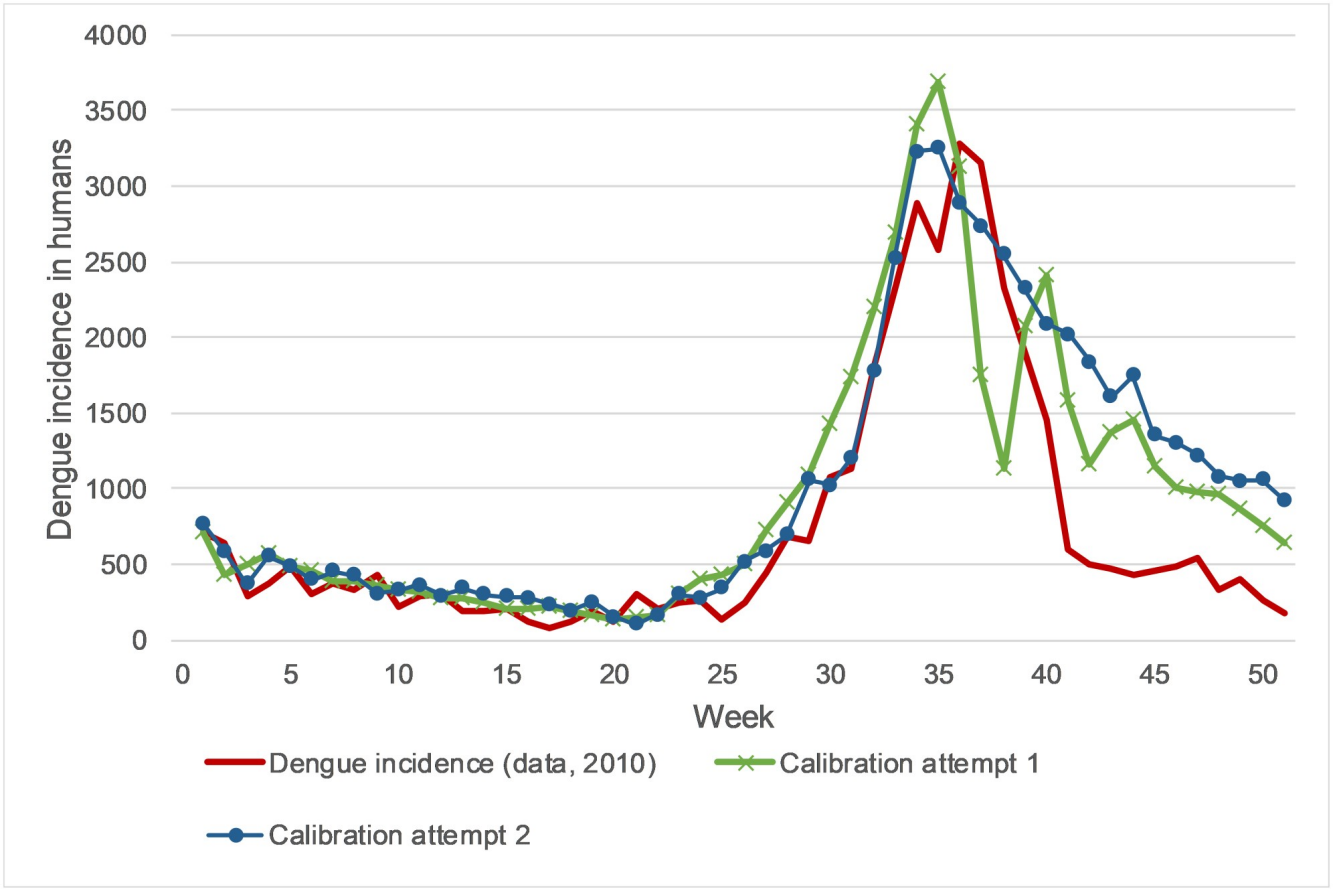
Calibration attempts. Fig 1 displays the results of the calibration attempts in comparison to the real-world data. Phase 1 and 2 are similar, using the two mosquito population adaptation techniques, while phase 3 results in different behavior.

Calibrating the third phase after week 37 was challenging because the developed system is inert and takes weeks to react to changes. Infected mosquitoes live for weeks, and humans are viraemic for several days after almost one week of incubation period. To stop the epidemics after week 37, two attempts were made (Fig 1, S3 File). Attempt 1 tries to reduce the mosquito population as soon as possible at the end of rainy season. Then, there are no newborn mosquitoes for 12 days. After that, the mosquito population has reached its defined number and a regular amount of mosquitoes per day are born. However, there are still a significant number of infected persons due to incubation period and viraemic period. The newborn mosquitoes biting these persons results in an unusually high prevalence among young mosquitoes. Consequently, the epidemic emerges again in week 39 (delayed by the mosquitoes’ incubation period), and a repeated smaller epidemic in week 43 appeared. In Attempt 2 a higher mosquito death rate was assumed, while still new mosquitoes are born. However, this results only in a slow decline because new mosquitoes always become infected quickly by humans.

**Fig 2:**
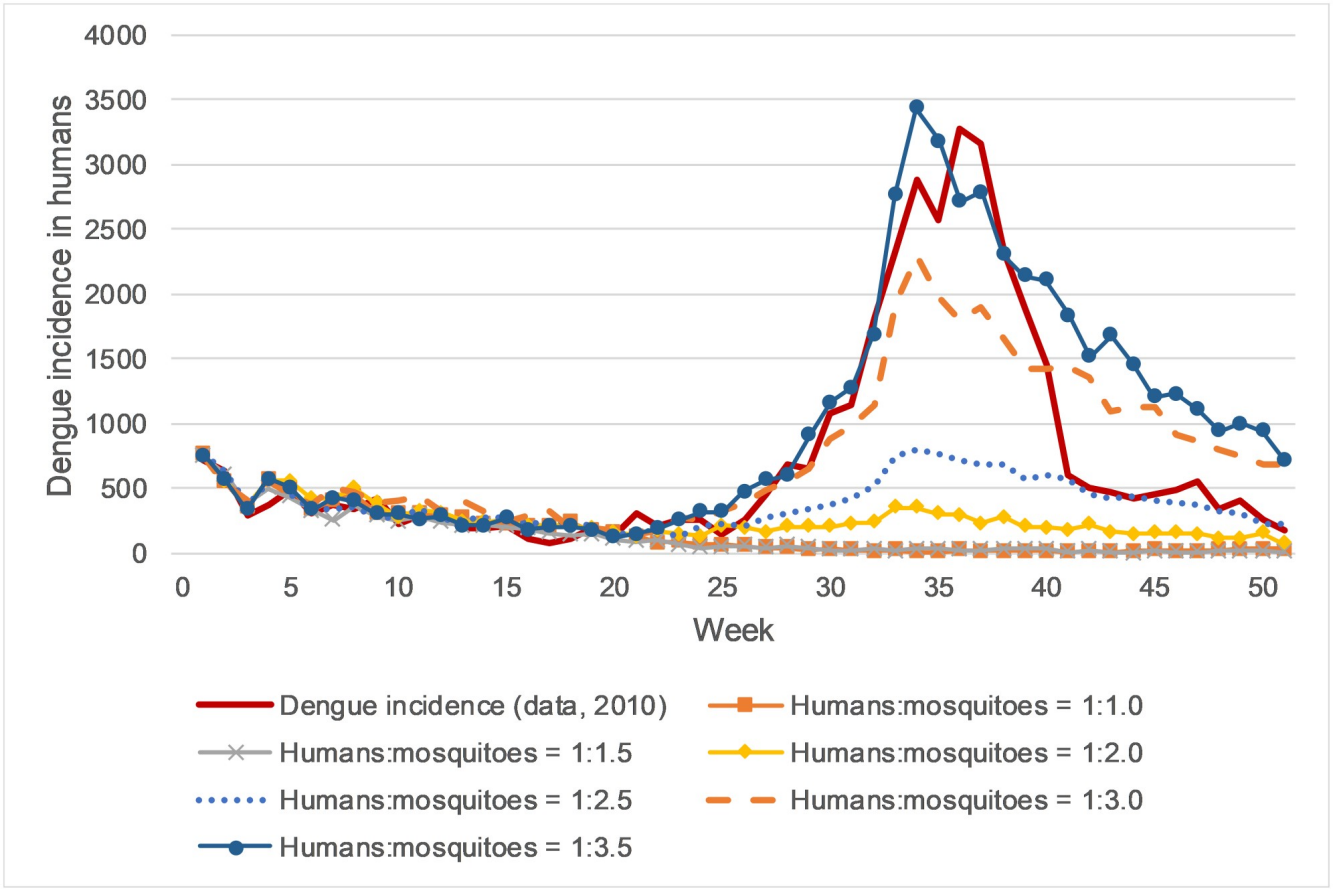
Timeline of the dengue incidence in humans in the year 2010. It compares the reported dengue cases to simulation results. 1:1.0, 1:1.5, 1:2.0, 1:2.5, and 1:3.0 refer to simulations with different human:mosquitoes ratios during raining season. The human:mosquito ratio describes the number of mosquitoes depending on the number of humans. This allows us to change the number of humans in the model without the need to change the number of mosquitoes, e.g., for scaling the model. The calibration result refers to the simulation with the calibrated parameters.

Finally, the model was calibrated following the second attempt of mosquito population adaptation. The best fit can be achieved when the mosquito population during rainy season rises to three times the human population, and declines to the same size as the human population after the rainy season. The resulting epidemic is presented in Table 1, Fig 2 and the supplementary material S4 File.

We validated the model according to the ISPOR-SMDM Good Modeling Practice Guidelines on several levels: (1) face validity (i.e., by public health experts, modeling experts), and (2) internal validation (debugging, consistency and plausibility checks) [51]. For internal validation, we also applied a simplified version of immersive face validation [52]. Immersive face validation involves observing a simulation from the perspective of a single agent. This provides different information than from aggregated model results, which typically represent the whole system. With immersive face validation, the behaviors of single agents are tested, ideally using a graphical 3D-representation of the system. Sophisticated forms of this validation even enable the programmer to perform actions with the agent during simulation time. In our dengue model, we used a simplified version of immersive face validation to observe, how often humans are bitten by mosquitoes, how many of them were infected and how often a mosquito bites viraemic and non-viraemic humans.

## Results

Fig 2 displays the incidence of infected people estimated by the calibrated model and the real-world data of Cebu City 2010. Predominantly we see a good fit of the curves. The simulated decline of the epidemics is slightly delayed.

The impact of potential mosquito control strategies on the number of new infections per week is displayed in Fig 2, it shows that a reduced human-mosquito ratio of 1:2.5 and 1:3 leads already to a substantial decrease of infected humans and smaller epidemics. Assuming a human mosquito ratio of 1:2 or smaller, the negative trend (i.e., decrease of new infections) during week 1 to 25 continues also during rainy season until the infection numbers reach zero. If a future intervention can reduce the ratio to 1:1 (i.e., number of mosquitoes does not increase during rainy season), the dengue epidemic eventually dies out. (see also S4 File.)

## Discussion

ABM is a dynamic modeling approach that is able to reproduce direct and indirect effects of interventions for communicable diseases and to replicate emerging behavior. We summarized major characteristics of ABM and illustrated in a case example how ABM can be applied to model a dengue outbreak and to evaluate the effect of potential mosquito control interventions differing in effectiveness.

Classical approaches for epidemic modeling often use ordinary differential equations or system dynamics, where aggregated variables represent the population of interest that is grouped by health and other attributes [18, 53–55]. Because of this aggregation level, model development, validation and sensitivity analyses tend to be faster as compared to individual-based models. However, the high abstraction level leads to less flexibility for subgroup analyses if this was not specified upfront (e.g., vaccination of all persons of a certain age that are frequently bitten by mosquitoes). In contrast, agent-based modeling simulates individuals. An individual-based simulation allows one to include and track patient characteristics and medical history, evaluation of subgroup specific interventions, calculating specific outcomes and distributions of these outcomes as well as modeling an open cohort. A specific distinguishing feature of ABM compared to other individual-based modeling approaches is that the behavior of agents and respective emerging behavior of the epidemics [56–58] can be captured. Changing the behavior of specific groups of agents is possible with less programming effort. In a compartmental model (e.g., system dynamics) this would require a restructuring of the compartments and respective underlying equations.

The flexibility of agent-based models supports the style of iterative modeling. Starting with a reduced and simplified model structure, more features (e.g. agent characteristics, potential disease pathways) can be added iteratively until the model complexity meets the needs of the respective (future) model applications. Reusability is an important issue because it can significantly reduce the effort for model development, and working with validated model parts increase both validity and credibility in the scientific community and for health policy decision makers. A disadvantage of individual-level ABM are longer conceptualization and model implementation times.

ABMs are constructed to reproduces an epidemic in a similar way as it happens in reality. The spreading process occurs based on agents’ behavior. Hence, the model gives insights into the mechanics of the underlying process, how the epidemic spreads among the population, and how system changes affect this spreading process, which eventually results in different outcomes. In particular, this allows to examine herd immunity and serotype shifts. Stochastic state transition models cannot produce an emerging behavior. They can only incorporate them if they are known for each situation, which is usually not possible for scenarios. In contrast to that, the agent-based model, where the epidemic emerges upon basic rules, automatically simulates these effects without incorporating them explicitly. Yet it is a matter of current research to find a commonly accepted definition and a standardized way for measurement of herd immunity and serotype shift, which makes it even harder to use them in models where they are explicitly required [59].

In ABM, it is technically even possible to trace back the mosquito that infected a person, when and where it was born, got infected, how many persons it infected before, and what it did afterwards until it died. Such information could support model validation and in particular the novel immersive face validation.

Compared to other modeling approaches, ABM also has limitations. ABM usually requires longer development time due to complexity. Because ABM are individual based, one faces longer simulation time, lack of analytical tractability, and challenges in parameterization [60]. For example, often there is a lack of studies on contact pattern or frequencies. Therefore, models require extensive calibration efforts for unknown parameters. However, these unknown parameter are made explicit and therefore they become accessible for discussions. Emergent behavior is both an advantage and a weakness of agent-based models. It allows simulating complex and even counterintuitive dynamics. However, the cause of wrong dynamics as a result of erroneous or incomplete agent’s behavior are hard to detect. Therefore, Klügl [61] presents a validation approach specifically for agent-based models that should help to detect such problems. Marshall et al. discuss a new dimension of sensitivity analyses, that is added and may be challenging in ABM: testing the assumptions about human behavior (e.g., “how people learn, how they disseminate information to their peers or families, and how they change their behavior in response to new information, incentives, or penalties”) in ABM [40]. Chattwahl and He [38] discuss challenges of probabilistic sensitivity analyses due to transmission and network-related parameters that are correlated. In addition, probabilistic sensitivity analysis can require long simulation time because of the combination of first- and second-order uncertainty. Value of information analysis to discover parameters of future research would also be computational very intense. Modeling an open cohort leads to the question of how outcomes are summarized. For example, within a cost-effectiveness analysis of vaccinations of newborns one could consider the outcome (e.g., health-related quality of life) for the entire population over a fixed time horizon or the outcome summarized for people that have at least a follow up time of ten years. Finally, as with all complex models, visualization of ABM with respect to the several modules or layers (development of population, disease, contacts) could be challenging.

Literature on ABM comprises technical tutorial papers with general applications [4, 37], simulating human systems [62], tutorial on economic evaluation using ABM [38], teaching ABM [37] and comparison of modeling approaches and guidance on model selection [4, 40, 41, 63]. In contrast to this existing literature, we provided a tutorial example on why and how to apply ABM for decision analysis in infectious diseases, and on limitations.

In principle, the concept of ABM is independent of any particular software. However, there are specific software packages that have been designed to be applied or to integrate ABM. Here, we name an exemplary selection of available software. Beyond several commercial products such as ANYLOGIC (The AnyLogic Company), there are open-source agent-based simulators such as MASON, which has also been used to simulate epidemics [64] and EpiSimS, an agent-based simulation engine for modeling the spread of disease in regions developed by Los Alamos National Laboratory in the United States [65]. Repast, an open-source toolkit developed at the University of Chicago, is a rather general framework for agent-based modeling that provides some basic functionality while the users have to implement the model themselves in JAVA, .net, or Python [66].

Technical model implementations do usually not follow strict guidelines or instructions. Instead, they require an algorithmic approach that heavily depends on the actual problem. Hence, existing ABM simulators often have shortcomings for specific models because they cannot consider all possible requirements. In these cases, the simulator must be extended by the user or one decides to implement the model from scratch in a general programming language. Thus, technical programming experience with the specific software is crucial for ABM modeling and advanced general computer programming skills are often necessary.

The case example of dengue epidemics is challenging because the disease is not directly transmitted from human to human but from humans to mosquitoes and mosquitoes to humans. This requires modeling of two different populations leading to complex dynamics. The agent-based dengue model illustrates how these complex dynamics can be reproduced to a great extent, but producing the exact emerging behavior would require increased model complexity.

In our modeling example, each day 20% of the mosquitoes die and are replaced by new mosquitoes. This requires deletion and creation of a large number of agents, which affects the computation performance. Thus, the runtime heavily depends on the actual implementation, on used data structures and RAM management. The model has been implemented using JAVA based on a framework developed in earlier projects [42]. A sample simulation of 365 days with the full population on an Intel^®^ Xeon^®^ 3.3 GHz processor runs in 9 minutes and 54 seconds and needs a maximum of 600 MB RAM during dry season and 1.3 GM during rainy season.

Our case example has the following major limitations. In the population model, aging, deaths, births, emigration and immigration of the human population are not considered. This assumption can be reasonable for short-term analyses. For future long-term analyses however, open cohort dynamics should be implemented. We did not consider medical history including prior dengue infections with a different serotype that impacts the severity of the disease. Available data to model past dengue infections are limited. With respect to the mosquito incidence, we did not model in detail regionality of breeding places and the growth of the mosquito population during rainy season. The mosquito population size and age distribution, and population dynamics such as growth and decline at the beginning and end of the rainy season have been widely calibrated due to a lack of specific data. The decline of the mosquito population at the end of the rainy season in the calibrated model was slightly slower than the decline displayed by the real world data. This gap is assumed to be caused by unknown biological processes in the mosquito population. For example, the cause of the declining population could be an increase in death rate or a decrease in birth rate. Therefore, the emergent behavior at the end of the rainy season could not be exactly estimated. A common challenge for epidemics also strikes our dengue example: epidemics often occur in yearly, seasonal outbreaks. However, these outbreaks are usually completely different from year to year. Epidemiologists often can only provide limited explanations; therefore the goodness of a model prediction of any upcoming seasonal epidemic is limited. The only sensible approach is to use as an example a single, well-known epidemic from the past to test the effects of interventions. Applying an epidemic model to another season usually requires a fresh calibration.

Finally, we did not include adverse effects (e.g., of repellents, resistance) on health-related quality of life or the system itself and costs for our demonstration purposes but cost-effectiveness analysis could be performed as previously shown [38].

Further interventions that may be considered are: preventing from being bitten by mosquitoes (e.g., by repellents) and vaccinations. Due to the flexibility of our modular ABM, vaccinations can be implemented with manageable effort. Only one additional attribute needs to be added that characterizes an immune person, and a structure that incorporates vaccination strategies. This is an excellent example for flexibility since it does not affect the rest of the model structure.

In our case example, calibration—in particular of the declining epidemics—can be improved. Reasons of the sudden and very steep decline in our case example are not fully understood by epidemiologists. Hence, implementation of the underlying processes is difficult and may require adding additional complexity to the model. In comparison to other studies, for example the Yucatán model published by Hladish, we see a wide range of similarities [22, 23]. In the Yucatán model, the mosquito population starts to grow in May and reaches its maximum in July, before it declines in September. The number of dengue cases starts increasing in July, reaches its maximum in October, and then slowly decreases until January. Our model could reproduce such a slow decrease of dengue cases. However, the dengue epidemic in the Philippines stops abruptly within three weeks. Due to the long delay between size of mosquito population and dengue epidemic, we could not fully reproduce the rapid decline.

Our model could be further improved with regard to regionality, seasonality, serotypes, patient history and adaptive behavior. Incorporating households and the detailed location where persons and mosquitoes live can, for example, support the evaluation of targeted (regional) vaccination strategies and regional measures to contain the mosquito population. Incorporating seasonality (i.e., the impact of rain seasons on mosquito growth) could improve estimated monthly health care demand and subsequently support planning of health care provision. To differentiate between the four dengue serotypes in the model could also improve the evaluation of vaccination strategies. Studies have shown that persons can only become resistant against their serotype of infection, and that they are more likely to develop severe symptoms during secondary infections [12, 30]. Therefore, vaccination against specific serotypes could also prevent severe symptoms of secondary infections which may be considered as an additional benefit in a cost-effectiveness analysis. The model could be refined to incorporate patient histories including previous infections or comorbidities to allow, for example, for predictions on the severity of the disease. Further advanced topics such as adaptive behavior (e.g., humans taking prevention against mosquito bites, mosquitos getting resistant against certain measures) could also be considered in decision analyses.

In general, for the application of ABM for infectious diseases, there are several open questions for further research with respect to model validation and uncertainty analysis. For model validation a novel face validation technique where a human expert checks systematically what an agent perceives and how it reacts (immersive face validation) deserve closer attention [52]. For sensitivity analysis, testing the assumptions about human behavior [41] and doing a probabilistic sensitivity analysis within a reasonable time and taking into account correlation between parameters remain challenging [38].

## Conclusions

ABM is a powerful tool to support decision-analyses for infectious diseases including the dynamics of infectious diseases and emerging behavior, and with a high level of reusability due to modularity. Conceptualizing an ABM, the level of required detail should carefully be considered based on research questions to define a reasonable complexity. The ability to include human behavior or the behavior of other agents makes assumptions explicit, accessible for discussion but may also lead to extensive calibrations due to a lack of data. The complexity of ABM and the underlying structure often requires longer modeling, validation and simulation time. Models can become very data intense. Probabilistic sensitivity analysis can be challenging because of required information on parameter correlation and long simulation time because of the combination of first- and second-order uncertainty. Our case example illustrates that intense calibration efforts may lead only to reasonable reproducibility of reality. The focus of future research should be on model calibration and validation.

## Supporting information

S1 File Dengue case data. Hospitalized dengue cases. Data about all hospitalized dengue cases of persons living in Cebu City in 2010.
(XLSX)

S2 File Dengue computations. Computations of parameters. Computations of dengue case numbers and parameters based on the dengue case data.
(XLSX)

S3 File Calibration attempts. The datasets containing the resulting epidemics of the two calibration attempts for mosquito population adaptation.
(XLSX)

S4 File Calibration results. The simulation results of the calibration attempts with different humans:mosquitoes ratios.
(XLSX)

S5 File Dengue model documentation. A detailed documentation of the dengue model.
(DOCX)
