# Supplementary material for: Why should we apply ABM for decision analysis for infectious diseases?—An example for dengue interventions
Source: PLoS One. 2019 Aug 27;14(8):e0221564. doi: 10.1371/journal.pone.0221564 (PMC6711507; doi:10.1371/journal.pone.0221564)
Supplement: S5 File — A detailed documentation of the dengue model. (DOCX) [file pone.0221564.s005.docx]

Appendix S5 Dengue Model Documentation

# Modular Model Overview

The underlying concept is simulating dengue epidemics as a result of interacting human and mosquito agents. Mosquitoes bite humans, and dengue can be transmitted in both directions: from infectious humans to susceptible mosquitoes and from infected mosquitoes to susceptible humans. Both humans and mosquitoes have sophisticated behavior concerning their age and life span, reproduction, biting behavior, and disease progression.

The model does not distinguish between serotypes and simulates only one serotype since there is evidence that a dengue outbreak is typically caused by a single serotype [1]. The issue of secondary infection cannot be applied since there is no recent data about prevalent dengue serotypes and immunity from prior infections in the Philippines or in Cebu City.

The agents are modeled in a modular way, based on the idea of a modular agent-based framework for epidemic modeling [2]. Agents are self-contained individuals, and the modular approach aims to make it easier and clearer to define attributes and behavior. The three main modules are Population, Contacts and Disease. Each module handles all relevant tasks for initializing agents and equipping each of them with the respective attributes and behavior. The population module creates human and mosquito agents with age, gender, reproduction and deaths. The contact module creates barangays and adds behavior to agents relevant for how mosquitoes bite persons. The disease module handles everything related to dengue: transmissions, infection progress and recovery. Another module that is more often used as an out-of-the-package utility is the Protocol module, which tracks agents and generate results.

Implementing the model this way has several benefits. By breaking down the program into different modules, code construction and readability become more straightforward since simpler tasks are easier to understand and manage. Modules that are self-contained and loosely coupled also improve the efficiency of development because they can be conveniently maintained, reused or changed. [2]

# Simulation

The model simulates over time with discrete time steps, which are atomic by definition and cannot be split up further. This requires a special handling of actions that happen simultaneously from a model’s point of view. Figure 1 presents simulation phases, which are divided in sub-phases. Together with the modular method, this is a well-structured approach that makes conceptualization, implementation and communication easier and prevents errors.

During the initialization phase, the entire model setup happens. This means that agents, both persons and mosquitoes, are created and are assigned attributes and behavior. Furthermore, necessary environment structures are constructed. In the first part of the simulation phase, the agents can perform their actions based on their behavior, like interacting with each other, but without changing any attributes. In the second part, agents can update their attributes based on previous happenings. This includes a sophisticated collision handling because actions cannot be ordered temporally but only logically. For example, if an agent becomes immune and gets infected during the same day, the immunization might overrule the infection. At the end of the simulation phase, the protocol module keeps track of states and changes of interest, as well as other relevant environmental information. The simulation phase is repeated for each time step. Finally, in the results phase, the simulation terminates, and the protocol module stores and analyzes all collected data in appropriate file formats.


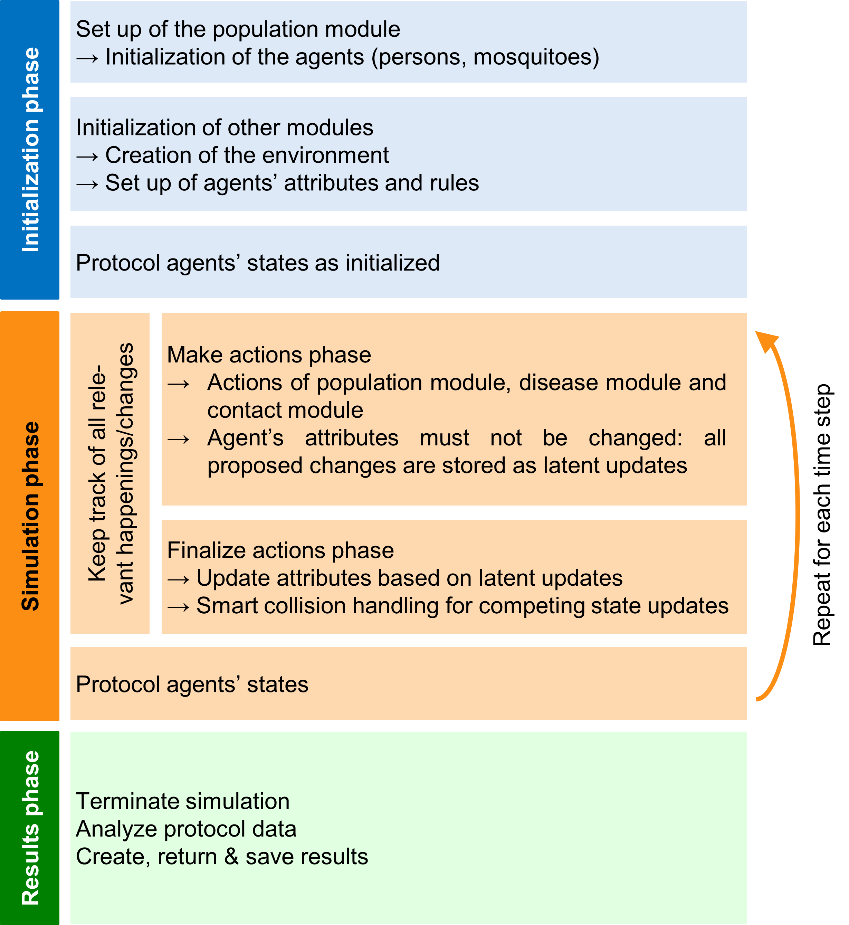


**Figure 1: Overview of the simulation phases**

# Population Module

## Human Population

For the human population, the module initializes persons, which possess the attributes age (in years) and gender. Due to an aimed simulation time of less than one year, the human population is modeled to be static. This means that nobody can leave or enter the system; hence there are no deaths, births, emigrants or immigrants. Distribution of age and gender in the human population is defined through parameters.

## Mosquito Population

The mosquito agents have an age (in days) and a gender as attributes. The difference from the human population is the short life span of mosquitoes, which lasts only for a few weeks. Since mosquitoes remain infected for the rest of their life and newborn mosquitoes are generally susceptible, it is crucial to model births and deaths accordingly. This change of generations allows valid representation of the level of infection in the mosquito population over the course of a year.

Besides a maximum age under [3], there is no information available about the age structure and survival probabilities of natural mosquito populations. Hence, we define that a mosquito dies each day with a given probability. If it survives day by day, it certainly dies when it reaches the defined maximum age. This results in an exponentially distributed age structure.

A female mosquito lays eggs at the end of each gonotrophic cycle. The number of eggs and breeding time are defined as parameters. The biting behavior of the gonotrophic cycle and detection of its end is modeled in the Contact module.

The mosquito population size is defined relative to the human population through a parameter for number of mosquitoes per person. It can be seen as a natural capacity for the mosquito population, dependent on the human population. This grants best flexibility. To simulate seasons, the capacity can change dynamically during simulation time. Maintaining a stable population size and adapting to new capacities require a sophisticated population control. The large number of eggs would result in rapid exponential growth that is highly implausible. Therefore, eggs can only hatch with a given probability; otherwise they die. This probability is recomputed every time step so that only a desired number of mosquitoes are born. There are different methods to achieve that. The first method simply allows enough births that the capacity is not exceeded if the population is stable or grows, and does not allow any births if the population should shrink. The second smooth method computes the births as deaths/(current population)⋅capacity. This term represents the number of births, which equals the number of deaths, in the balanced state of the capacity, no matter if the current population is already in this state or not. While the first method allows a rapid adaptation to new capacities, the second method provides a slower and more natural approach.

# Contact Module

Dengue transmission happens when a susceptible person gets bitten by an infected mosquito (carrier). Transmissions from person to person or mosquito to mosquito are not possible. This module handles everything that finally leads to bites, which are the only relevant interactions between agents.

Barangays are the smallest administrative units in the Philippines. They are modeled as logical units that can hold human and mosquito agents. Parameters define the number of barangays and their population. During initialization, barangays are created, and persons and mosquitoes are assigned randomly to one barangay with respect to their sizes and to the person-dependent mosquito capacity. Hence, each person has an assigned home barangay.

During simulation, persons can visit other barangays. In each time step, they randomly choose a number of other barangays they visit, additional to their home barangay. Mosquitoes only stay in their home barangay.

Female mosquitoes have to bite persons according to their gonotrophic cycle. The length of the gonotrophic cycle, the required amount of blood per gonotrophic cycle and the average amount of blood per bite are defined as parameters. A female mosquito aims to suck the same amount of blood each day. Hence, each day it bites so many people within its home barangay, until the amount is reached. For each bite, it randomly chooses one person among all resident and visiting persons.

# Disease Module

## Disease States of a Mosquito

The mosquito can be in one of the following states - susceptible, extrinsic incubating or infected. At birth, a mosquito is always susceptible. When a susceptible mosquito bites a person with a viraemic disease state, a transmission can happen with a given probability. If the transmission is successful, the mosquito undergoes an extrinsic incubation period of a given length, after which is becomes infected. The mosquito remains infected throughout its lifetime. Only mosquitoes in the infected state can transmit dengue to humans. Besides this, the infection does not affect the mosquito. Figure 2 visualizes the disease states of a mosquito.


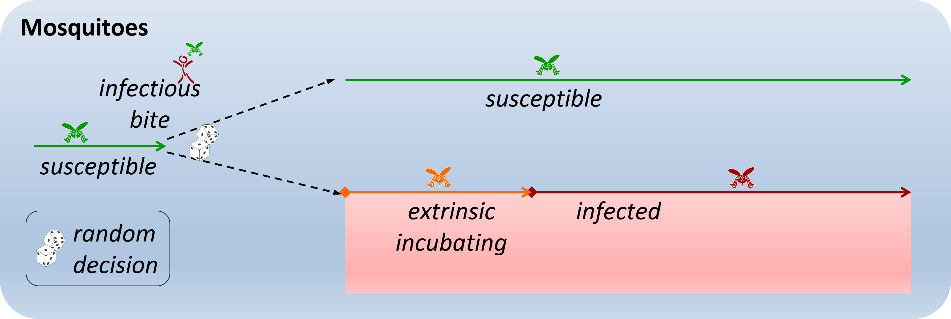


Figure 2: Disease states and state changes of a mosquito

## Disease States of a Person

A person can have one of two health states: infection and symptoms, which are schematically shown in Figure 3.


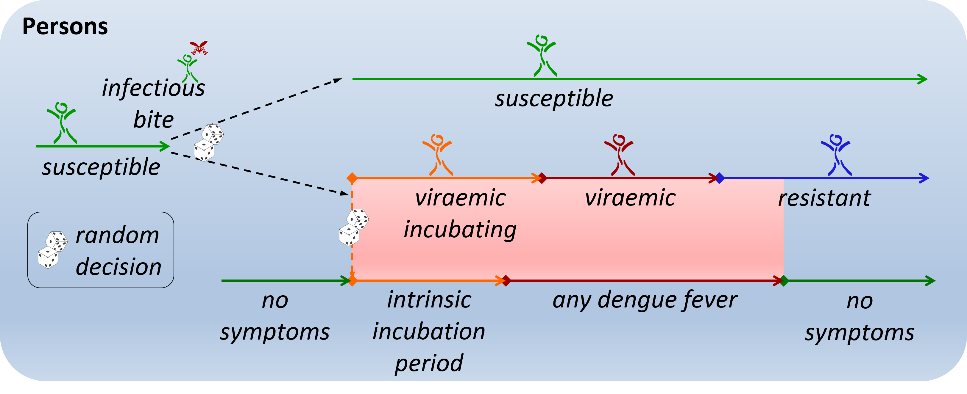


Figure 3. Disease states and state changes of a person

A person is susceptible if the person does not carry the dengue virus. When the person is bitten by an infected mosquito, a transmission can happen with a given probability. After a successful transmission, the person becomes “intrinsic incubating” for a given period, then viraemic and then resistant. A person stays resistant for the rest of the simulation time and cannot get infected again. However, only viraemic persons are able to transmit the virus when they are bitten by a susceptible mosquito.

Upon a successful transmission, the person randomly decides to develop symptoms or to remain without symptoms. If the person decides to develop symptoms, the person undergoes an intrinsic incubation period first, which should not be mistaken for the extrinsic incubation period of mosquitoes. After that, the person develops one of the fevers DF, DHF or DSS. Finally, the person goes back to the “no symptoms” state. This progress of symptoms starts with a transmission but runs independently from the infection progress.

# References

1. Halstead SB. Dengue. Lancet. 2007;370: 1644–1652. doi:10.1016/S0140-6736(07)61687-0

2. Miksch F, Urach C, Einzinger P, Zauner G. A Flexible Agent-Based Framework for Infectious Disease Modeling. In: Linawati, Mahendra M, Neuhold E, Tjoa Am, You I, editors. Information and Communication Technology. Springer Berlin Heidelberg; 2014. pp. 36–45. Available: http://dx.doi.org/10.1007/978-3-642-55032-4_4

3. Southwood TR, Murdie G, Yasuno M, Tonn RJ, Reader PM. Studies on the life budget of Aedes aegypti in Wat Samphaya, Bangkok, Thailand. Bull World Health Organ. 1972;46: 211–226.
